# Early intestinal ultrasound findings predict remission and treatment response at 1 year in pediatric Crohn’s disease

**DOI:** 10.1093/ecco-jcc/jjag036

**Published:** 2026-03-20

**Authors:** Alexandra S Hudson, Daniela M Isaac, Henry Ma, Anna Kuc, Matthew W Carroll, Eytan Wine, Hien Q Huynh

**Affiliations:** Edmonton Pediatric IBD Clinic (EPIC), Division of Pediatric Gastroenterology and Nutrition, Department of Pediatrics, University of Alberta, Edmonton, Alberta, Canada; Edmonton Pediatric IBD Clinic (EPIC), Division of Pediatric Gastroenterology and Nutrition, Department of Pediatrics, University of Alberta, Edmonton, Alberta, Canada; Edmonton Pediatric IBD Clinic (EPIC), Division of Pediatric Gastroenterology and Nutrition, Department of Pediatrics, University of Alberta, Edmonton, Alberta, Canada; Edmonton Pediatric IBD Clinic (EPIC), Division of Pediatric Gastroenterology and Nutrition, Department of Pediatrics, University of Alberta, Edmonton, Alberta, Canada; Edmonton Pediatric IBD Clinic (EPIC), Division of Pediatric Gastroenterology and Nutrition, Department of Pediatrics, University of Alberta, Edmonton, Alberta, Canada; Edmonton Pediatric IBD Clinic (EPIC), Division of Pediatric Gastroenterology and Nutrition, Department of Pediatrics, University of Alberta, Edmonton, Alberta, Canada; Edmonton Pediatric IBD Clinic (EPIC), Division of Pediatric Gastroenterology and Nutrition, Department of Pediatrics, University of Alberta, Edmonton, Alberta, Canada

**Keywords:** intestinal ultrasound, pediatric, bowel wall

## Abstract

**Background:**

Intestinal ultrasound (IUS) in pediatric IBD is increasing, but evidence remains limited compared with adults. This study aimed to evaluate the relationship between IUS and clinical, biochemical, and endoscopic measures in pediatric Crohn’s disease patients over 1 year, and to assess its ability to predict treatment response and remission.

**Methods:**

Pediatric patients with suspected inflammatory bowel disease were prospectively enrolled and assessed over 1 year. Bowel wall thickness (BWT) and three validated IUS scores were calculated. Remission was combined clinical/biochemical/endoscopic remission. Treatment response was not needing therapy escalation and achieving remission by 1 year.

**Results:**

Sixty-one patients, median age 12.3 years (IQR 10.3-14.7; range 6-17), were included. IUS correlated moderately to strongly with endoscopy (rho 0.43-0.70). Thicker terminal ileum BWT was associated with increased likelihood of ileocecal resection (*n* = 7, 12%) (OR = 5.85, 95% CI 1.29-26.47, *P* < .05). Patients’ thickest bowel segment became significantly thinner at 1 month (*P* < .05). Thicker BWT at 6 months was associated with a decreased likelihood of 1-year remission (OR = 0.34; 95% CI 0.16-0.74, *P* = .006). BWT ≤ 2.8 mm at 6 months predicted treatment response with high sensitivity (73%) and specificity (84%). BWT ≤ 2.5 mm at 1 year predicted remission with high sensitivity (72%) and specificity (90%).

**Conclusions:**

IUS correlated strongly with endoscopy in pediatric Crohn’s disease over 1 year, with increased BWT being associated with a higher risk of ileocecal resection and not achieving remission. Thickened (inflamed) bowel became significantly thinner as soon as 1 month. BWT in pediatric patients in remission was significantly lower (≤2.5 mm) than in adults (≤3–4 mm).

## 1. Introduction

Gastroenterologists are monitoring inflammatory bowel disease (IBD) patients more closely and more often than ever before, with the STRIDE II guidelines^1^ highlighting the multiple patient and disease targets (short, medium, and long term).^2^ Intestinal ultrasound (IUS) has become well established as an IBD assessment and monitoring tool in Europe and is growing exponentially in popularity in North America. It has been shown to correlate with inflammation activity (endoscopic, biochemical, and clinical)^3^  and is more easily repeated than magnetic resonance enterography (MRE) and endoscopy. Both adult^4^  and pediatric^5^ patients (including their caregivers) have been found to prefer IUS to the other available IBD assessment modalities that gastroenterologists currently repeatedly use.

The majority of the available literature on the use of IUS in IBD is limited to adult patients.^6^ In pediatrics, most studies are retrospective, cross-sectional, and limited to small numbers of patients with scarce long-term data. Total bowel wall thickness (BWT) as well as composite IUS scores (combining BWT, Doppler signal, inflammatory fat, among other parameters) have been the main measurements of interest but with mixed data. BWT reduction below 1.9-3.1 mm has been reported for pediatric bowel with no endoscopic inflammation or in endoscopic remission, at various time points but as early as 8 weeks.^7–9^ In pediatrics it is still unclear how total BWT and IUS scores compare to repeated clinical, biochemical, and endoscopic assessments over a longer period of time.

Given that the incidence and prevalence of IBD is rising worldwide, with pediatrics being the fastest growing patient group, this is an important patient group to continue to assess. Particularly in pediatrics, IUS is attractive as it does not require the general anesthesia that endoscopy needs, and is especially helpful for young children who cannot complete an unsedated MRE scan or who may have a fear of blood work. IUS may be a particularly helpful tool in Crohn’s disease, given its transmural inflammatory process. The primary objective of this study was therefore to describe longitudinal IUS findings and its relationships with markers of IBD activity (clinical, biochemical, and endoscopic) in pediatric patients with newly diagnosed Crohn’s disease up to 1 year post-diagnosis. We also aimed to identify which early IUS findings (1, 3, and 6 months post-diagnosis) could prospectively predict remission (combined clinical, biochemical, and endoscopic) at 1 year.

## 2. Materials and methods

### 2.1. Procedure

Research ethics board approval was sought prior to study initiation. Patients (0-18 years) with suspected IBD were prospectively enrolled through the Edmonton Pediatric IBD Clinic from September 2019 to January 2023, and followed until January 2024. Those diagnosed with Crohn’s disease and had completed the IUS at 6 months post-diagnosis were included in this analysis.

Patients underwent assessments at baseline, 1 month, 3 months, 6 months, and 1 year. Each assessment included IUS, clinical assessments, blood work, and fecal calprotectin (FCP). Endoscopy was done at baseline (after the baseline IUS) and between 6 months and 1 year. MRE was completed at baseline (after IUS). MRE strictures were based on the radiology report describing intestinal lumen narrowing with upstream dilatation suggestive of a stricture. Blood work included C-reactive protein (CRP), erythrocyte sedimentation rate (ESR), albumin, and hemoglobin. The timing and type of IBD surgery, if needed, was noted. Remission was defined as: FCP < 250 mg/kg, CRP < 4 mg/L, weighted Pediatric Crohn’s Disease Activity Index (wPCDAI) < 12, and no upcoming surgery, as well as Simple Endoscopic Score for Crohn’s Disease (SES-CD) ≤ 2 for each bowel segment (included if endoscopy was done at that time point). Presence or absence of remission was documented at each assessment. Treatment response was defined as not requiring therapy escalation at all subsequent time points and achieving remission by 1 year. Therapy escalation was defined as giving a steroid course, changing from a non-biologic medication to a biologic medication, or changing biologic medications.

### 2.2. Intestinal ultrasound and image acquisition

Intestinal ultrasounds were conducted in the Pediatric Gastroenterology clinic at the time of the comprehensive clinic visit using a Philips EPIQ 5q machine (linear L12-5 probe). One of two pediatric gastroenterologists (H.H., D.M.I.) completed the scans, International Bowel Ultrasound (IBUS) certified (H.H., D.M.I.) as well as an IBUS certified trainer (H.H.) with over 1000 scans completed. BWT was measured longitudinally and cross-sectionally in millimeters, recording two or more measurements (minimum 1 cm or 90° apart) in the most severe/affected part of the terminal ileum, and proximal and distal sections of each colonic bowel segment (ascending, transverse, descending, and sigmoid colons). The rectum was excluded as it is difficult to accurately visualize. Each bowel segment had its measurements averaged. The thickest BWT for that segment was also noted. The Doppler setting for flow was ±5 cm/s, and hyperemia was recorded for each bowel segment. Hyperemia was scored with the modified Limberg score: 1 = short signals (corresponding to mild hyperemia or 2-5 vessels/cm^2^), 2 = long signals inside the bowel (corresponding to moderate hyperemia or >5 vessels/cm^2^), 3 = long signals inside and outside the bowel (corresponding to moderate hyperemia or >5 vessels/cm^2^).^10^ Other information collected included presence/absence of strictures, excess lymph nodes, fat proliferation, free fluid, haustration, and bowel wall stratification. Strictures were defined as luminal narrowing with upstream bowel dilatation ≥2.5 cm.^11^

### 2.3. Materials

Clinical assessments used the wPCDAI, defining remission (<12.5), mild (12.5-40), moderate (>40-57.5), and severe (>57.5) clinical disease activity. Medications used for induction therapy and maintenance therapy, as well as type, timing, and reason for medication changes were collected.

Endoscopic assessments used the SES-CD^13^ for each bowel segment (terminal ileum, right colon, transverse colon, left colon, rectum). The maximum segment score is 12, with a maximum total score of 60. The SES-CD score for this study analysis was modified to exclude the rectum to allow for correlation between endoscopy and IUS (which excludes the rectum in this study), resulting in a maximum total modified score of 48. A maximum score of 12 was assigned to the terminal ileum if the ileocecal valve was strictured and the terminal ileum was unable to be visualized.

Crohn’s disease intestinal ultrasound scores published in the literature at the time of data analysis were used on the prospective collected IUS data, scoring the terminal ileum and right/transverse/left colon segments and a total score. This included the Simple Ultrasound Activity Score for Crohn’s Disease^14^ (SUS-CD), scoring each segment’s BWT from 0 to 3 (0 = <3 mm, 1 = 3-4.9 mm, 2 = 5-7.9 mm, and 3 = ≥8 mm) and colour Doppler score from 0 to 2 (0 = none, 1 = 2-5 vessels/cm^2^, 2 = >5 vessels/cm^2^). The maximum segment score was 5 and maximum total score was 20. The second score used was the Simple Pediatric Activity Ultrasound Score (SPAUSS),^15^ scoring BWT (1-3.99 mm = 1, 4-6.99 mm = 4, ≥7 mm = 6), Doppler signal (mild hyperemia = 1, moderate/severe hyperemia = 2), and inflammatory fat (mild = 1, moderate/severe = 6), for a total possible segment score of 14 and total score of 56. The third IUS score used was the IBUS-SAS,^10^ which calculates BWT, inflammatory fat (1 = uncertain, 2 = present), Doppler signal (1 = short signals, 2 = long signals inside the bowel, 3 = long signals inside and outside the bowel), and bowel wall stratification (BWS) (1 = uncertain, 2 = focal, 3 = extensive), for a bowel segment calculation of 4 × BWT + 15 × fat +7 × Doppler + 4 × BWS. This score therefore has no total possible segment score or total score.

### 2.4. Statistical analyses

Descriptive statistics were utilized to calculate median values with 25th-75th interquartile ranges (IQR) for continuous variables, and frequency with percentages for categorical variables. Spearman’s rank (rho) was used to assess correlations for IUS total scores and BWT of the thickest bowel segment with endoscopic, clinical, and biochemical disease activity markers. Non-parametric (Mann-Whitney U, Wilcoxon Signed Ranks) tests were used to compare groups. Binary logistic regression models were performed to assess the effect of IUS, clinical, biochemical, and endoscopic factors on the likelihood of achieving remission at 1 year as well as needing intestinal surgery by 1 year. Receiver operating characteristic (ROC) analyses were completed to assess the sensitivity and specificity of BWT in differentiating between those who achieved remission by 1 year with no major therapy escalation or change. Youden’s index was calculated to help inform the optimal BWT cut-offs for remission. All analyses were done in SPSS v.20.0.0 (IBM^®^ SPSS© Statistics, New York, NY, USA) and a *P* value of≤.05 was considered statistically significant. All analyses were two-sided.

## 3. Results

### 3.1. Patients

Sixty-one patients (*n* = 41/61, 67% male) with a median age of 12.3 years (IQR 10.3-14.7, range 6-17) were included. Baseline patient characteristics are given in Table 1. Baseline disease activity was moderate based on clinical (median wPCDAI 55, IQR 40-78), endoscopic (median SES-CD 13, IQR 9-21), and biochemical parameters (Table 1). The most common disease distribution was ileocolonic (*n* = 32/61, 53%). At baseline, 21% of patients (*n* = 13/61) had stricturing disease and 23% (*n* = 14/61) had perianal disease. Growth delay was identified in 43% (*n* = 26/61) of all newly diagnosed patients. Exclusive enteral nutrition (*n* = 26/61, 43%), followed by anti-tumor necrosis factor (TNF) alpha biologic (*n* = 21/61, 34%), were the two most common induction therapy choices (Table 1).

**Table 1.** Patient (*n* = 61) baseline characteristics.

Characteristic	Frequency (percentage) or median [25th-75th interquartile range]
**Age, years**	12.3 [10.3-14.7]
**Sex**	
** Male**	41 (67)
** Female**	20 (33)
**BMI z-score, kg/m^2^**	−0.93 [−2.6 to 0]
**wPCDAI^10^ score**	55 [40-78]
**SES-CD^12^ total score**	13 [9-21]
**SUS-CD^13^ total score (maximum score 20)**	4 [3-8]
**SPAUSS^14^ total score (maximum score 56)**	15 [10-19]
**IBUS-SAS^15^ total score (no maximum score)**	107 [88-139]
**Paris classification**	
** A1a (0 to <10 years)**	16 (26)
** A1b (10 to <17 years)**	45 (74)
** L1 (distal ⅓ ileum ± limited cecal disease)**	17 (28)
** L2 (colonic)**	11 (18)
** L3 (ileocolonic)**	32 (53)
** L4a (upper disease proximal to ligament of Treitz)**	28 (46)
** L4b (upper disease distal to ligament of Treitz and proximal to distal ⅓ of ileum)**	19 (31)
** B1 (non-stricturing, non-penetrating)**	47 (77)
** B2 (stricturing)**	13 (21)
** B3 (penetrating)**	0 (0)
** B1 and B2 (stricturing and penetrating)**	1 (2)
** p (perianal disease)**	14 (23)
** G1 (growth delay)**	26 (43)
**Biochemical markers**	
** CRP, mg/L**	40 [23-76]
** ESR, mm/h**	33 [18-44]
** FCP, µg/g**	1539 [982-3054]
** Albumin, g/L**	29 [25-34]
** Hemoglobin, g/L**	111 [101-118]
**Baseline induction therapy choice**	
** Exclusive enteral nutrition**	26 (43)
** Anti-TNF biologic (infliximab or adalimumab)**	21 (34)
** Steroids (intravenous or oral)**	9 (15)
** Crohn’s disease exclusion diet**	4 (7)
** Methotrexate**	1 (2)
**Maintenance therapy at 12 months post-diagnosis**	
** Anti-TNF biologic (infliximab or adalimumab)**	42 (69)
** Methotrexate**	11 (18)
** Ustekinumab**	2 (3)
** Crohn’s disease exclusion diet**	2 (3)
** Azathioprine**	2 (3)
** Vedolizumab**	1 (2)
** Mesalamine**	1 (2)

Abbreviations: BMI, body mass index; wPCDAI, weighted Pediatric Crohn’s Disease Activity Index; SES-CD, Simple Endoscopic Score for Crohn’s Disease; SUS-CD, Simple Ultrasound Activity Score for Crohn’s Disease; SPAUSS, Simple Pediatric Activity Ultrasound Score; IBUS-SAS, International Bowel Ultrasound Segmental Activity Score; CRP, C-reactive protein; ESR, erythrocyte sedimentation rate; FCP, fecal calprotectin; TNF, tumor necrosis factor.

### 3.2. Intestinal ultrasound

**Figure 1.**
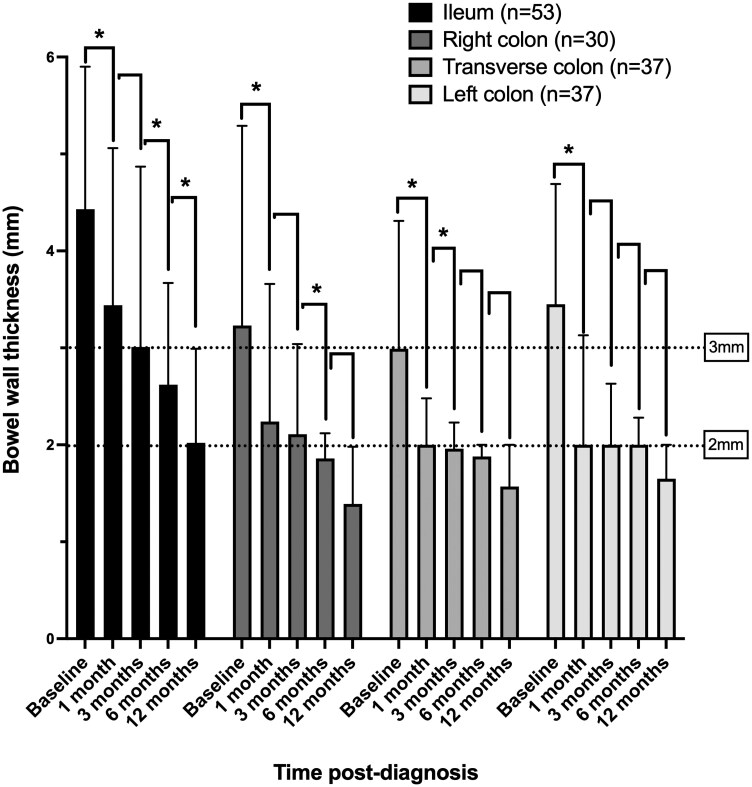
(A) Median bowel wall thickness (BWT) for each bowel segment at each time point. For BWT, only segments with a BWT of at least 2 mm at baseline were included, to demonstrate the trend in baseline abnormal segments only. Error bars represent the 25th to 75th interquartile ranges. *P < .05 was significant.

All patients (*n* = 61/61, 100%) completed up to the 6-month IUS, and 89% (*n* = 54/61) also completed a 1-year IUS. For those who had a bowel segment BWT of ≥2 mm at baseline (therefore excluding normal bowel), all median IUS BWT became significantly thinner in all bowel segments over time, as early as 1 month (Figure 1). At baseline and each subsequent time point, the terminal ileum median BWT remained thicker than colonic median BWT (Figure 1).

For the thickest bowel segment on baseline IUS for all patients, median improvement of BWT was −0.89 mm (IQR −2.72 to +0.12) at 1 month, −1.33 mm (IQR −2.63 to −0.27) at 3 months, −1.8 mm (IQR −3.5 to −0.53) at 6 months, and −2.19 mm (IQR −4.72 to −1.07) by 1 year. This was significantly thinner than the baseline thickest bowel segment as early as 1 month (*P* < .001). This translated to a median percentage reduction in BWT by −16% (IQR −43 to +3), −27% (IQR −43 to −4), −43% (IQR −60 to −15), and −45% (IQR −67 to −25), respectively.

For all patients, the median IUS total scores (SUS-CD, SPAUSS, and IBUS-SAS) also significantly improved from baseline to 12 months (*P* < .05). At baseline, median total scores were SUS-CD 4/20 (IQR 3-8), SPAUSS 15/56 (IQR 10-19), and IBUS-SAS 107 (IQR 88-139). By 1 year, median total IUS scores had dropped to SUS-CD 0/20 (IQR 0-1), SPAUSS 4/56 (IQR 4-5), and IBUS-SAS 30 (IQR 24-41).

### 3.3. IUS correlations to endoscopy

Over 1 year, IUS correlated most strongly with endoscopy, compared to clinical and biochemical markers of disease activity (Table 2). Correlations between median BWT and corresponding endoscopy for a bowel segment were significantly positive: terminal ileum rho = 0.74 (strong correlation), right colon rho = 0.39 (weak correlation), transverse colon rho = 0.40 (moderate correlation), and left colon rho = 0.54 (moderate correlation) (all *P* < .05). All three IUS total scores also significantly correlated with endoscopy total scores: moderate positive correlations to baseline endoscopy and moderate/strong positive correlations to follow-up endoscopy at 6 months to 1 year (Table 2).

**Table 2.** Spearman rho correlation between three intestinal ultrasound total scores and endoscopy, clinical, and biochemical assessments at each time point.

	Endoscopy (SES-CD total score)^b^	wPCDAI	Fecal calprotectin	CRP	ESR
**Baseline IUS (*n*** = **61/61, 100%)**	*n* = 61/61, 100%	*n* = 61/61, 100%	*n* = 57/61, 93%	*n* = 61/61, 100%	*n* = 57/61, 93%
** Thickest segment BWT^a^**	NA	0.06	0.29*	0.34*	0.04
** SUS-CD total**	0.44*	0.28*	0.28*	0.45*	0.48*
** SPAUSS total**	0.45*	0.21	0.29*	0.42*	0.34*
** IBUS-SAS total**	0.43*	0.25*	0.33*	0.43*	0.46*
**1-month IUS (*n*** = **55/61, 90%)**	NA	*n* = 61/61, 100%	*n* = 37/61, 61%	*n* = 57/61, 93%	*n* = 57/61, 93%
** Thickest segment BWT^a^**	NA	0.30*	0.29	0.30*	0.23
** SUS-CD total**	NA	0.30*	0.32	0.31*	0.28*
** SPAUSS total**	NA	0.38*	0.26	0.33*	0.25
** IBUS-SAS total**	NA	0.33*	0.32	0.32*	0.30*
**3-month IUS (*n*** = **56/61, 92%)**	NA	*n* = 57/61, 93%	*n* = 43/61, 70%	*n* = 60/61, 98%	*n* = 58/61, 95%
** Thickest segment BWT^a^**	NA	0.27*	0.37*	0.59*	0.42*
** SUS-CD total**	NA	0.28*	0.27	0.49*	0.39*
** SPAUSS total**	NA	0.32*	0.26	0.54*	0.39*
** IBUS-SAS total**	NA	0.27*	0.24	0.44*	0.30*
**6-month IUS (*n*** = **61/61, 100%)**	*n* = 16/61, 26%	*n* = 59/61, 97%	*n* = 51/61, 84%	*n* = 60/61, 98%	*n* = 57/61, 93%
** Thickest segment BWT^a^**	NA	0.41*	0.48*	0.48*	0.38*
** SUS-CD total**	0.70*	0.47*	0.54*	0.38*	0.33*
** SPAUSS total**	0.64*	0.56*	0.58*	0.43*	0.43*
** IBUS-SAS total**	0.50*	0.60*	0.59*	0.45*	0.44*
**One-year IUS (*n*** = **54/61, 89%)**	*n* = 30/61, 49%	*n* = 58/61, 95%	*n* = 54/61, 89%	*n* = 60/61, 98%	*n* = 58/61, 95%
** Thickest segment BWT^a^**	NA	0.36*	0.51*	0.25	0.47*
** SUS-CD total**	0.69*	0.53*	0.50*	0.15	0.53*
** SPAUSS total**	0.62*	0.43*	0.61*	0.22	0.41*
** IBUS-SAS total**	0.58*	0.43*	0.56*	0.22	0.39*

* *P*-value < .05 was significant.

a Thickest BWT was the BWT of the most inflamed (thickest) bowel segment (from terminal ileum, right/transverse/descending/sigmoid colon) for the patient.

b Excludes rectum.

Abbreviations: NA, not applicable; wPCDAI, weighted Pediatric Crohn’s Disease Activity Index; SES-CD, Simple Endoscopic Score for Crohn’s Disease; SUS-CD, Simple Ultrasound Activity Score for Crohn’s Disease; SPAUSS, Simple Pediatric Activity Ultrasound Score; IBUS-SAS, International Bowel Ultrasound Segmental Activity Score; CRP, C-reactive protein; ESR, erythrocyte sedimentation rate; FCP, fecal calprotectin.

### 3.4. IUS correlations to clinical assessment and biochemical markers

IUS correlated more weakly to the wPCDAI assessments, with weak positive correlations at baseline, 1 month, and 3 months (Table 2). These correlations became moderately positive correlations at 6 months and 1 year. There were no strong correlations between IUS (BWT or scores) and wPCDAI.

IUS correlated to biochemical markers (FCP, CRP, ESR) similarly to its clinical correlations (wPCDAI), with positive weak to moderate correlations over the year (Table 2). At certain time points IUS did not correlate at all to FCP (1 month) or CRP (1 year). Median clinical and biochemical parameters for all patients are given in Table S1.

### *3.5. BWT and remission at 1* *year*

**Figure 2.**
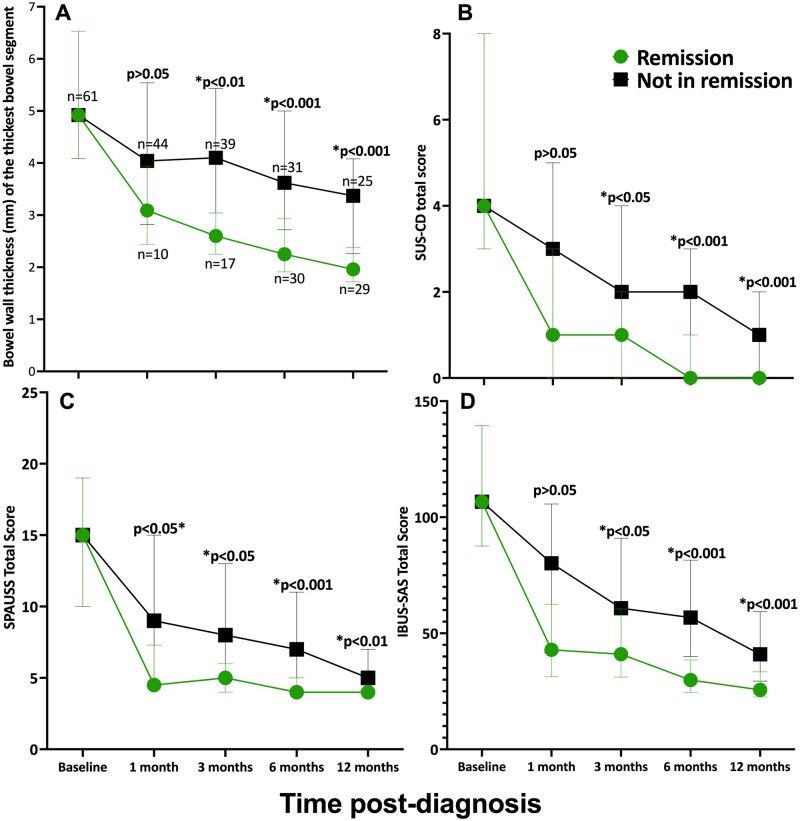
(A) Median bowel wall thickness (BWT) of the thickest bowel segment, (B) SUS-CD total score, (C) SPAUSS total score, and (D) IBUS-SAS total score, separated by those in remission (FCP < 250 mg/kg, CRP < 4 mg/L, wPCDAI < 12, no upcoming surgery, and SES-CD score ≤ 2 for each bowel segment) vs not. Error bars represent the 25th to 75th interquartile ranges. *P* < .05 was significant. There were patients with missing intestinal ultrasound (IUS) at 1 month (*n* = 7), 3 months (*n* = 5), and 1 year (*n* = 7). All patients (*n* = 61) had IUS at baseline and 6 months. SUS-CD, Simple Ultrasound Activity Score for Crohn’s Disease; SPAUSS, Simple Pediatric Activity Ultrasound Score; IBUS-SAS, International Bowel Ultrasound Segmental Activity Score.

Comparing those in remission to those not in remission at each time point, BWT of the thickest bowel segment became significantly thinner for those in remission as soon as 3 months post-diagnosis, which was sustained at all subsequent time points (*P* < .001 to *P* < .05) (Figure 2A). The median BWT for those in remission improved to below 3 mm (adult IBD remission cut-off^1^) by 3 months and to below 2 mm by 1 year (Figure 2A).

Similarly, all three intestinal ultrasound scores became ­significantly lower for those in remission as soon as 3 months post-diagnosis (*P* < .05) **(**Figure 2B-D**)**. The SPAUSS total score was also significantly lower for those in remission at the 1-month mark (*P* < .05) **(**Figure 2C). Patients in remission by 1 year had a significantly greater reduction in absolute number (in mm) and percentage (%) of their thickest bowel segment BWT over the 1 year: −2.52 mm (IQR −5.05 to −1.79) and −59% (IQR −72 to −44) vs −1.29 mm (IQR −3.55 to −0.20) and −27% (IQR −56 to −4) (*P* < .05).

A binary logistic regression analysis was performed to identify the effects of IUS, clinical, and biochemical assessments (at 1, 3, and 6−months) on the likelihood of achieving remission at 1 year. The 6-month analysis (χ^2^(5) = 17.39, *P* = .004) identified that a higher BWT of the thickest bowel segment at 6 months was significantly associated with a decreased likelihood of remission at 1 year (odds ratio [OR] = 0.34; 95% confidence interval [CI] 0.16-0.74, *P* = .006). Six-month FCP, CRP, and wPCDAI were not significantly associated with achieving remission at 1 year. BWT, FCP, CRP, and wPCDAI at 1 and 3 months were also not significant.

**Figure 3.**
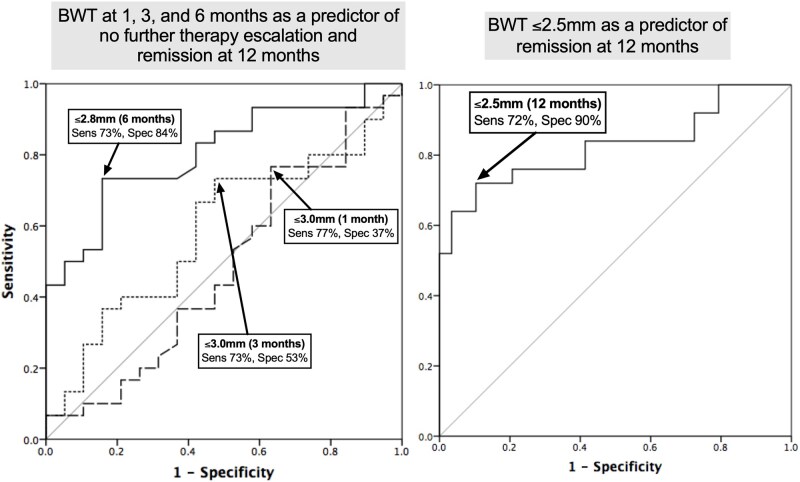
ROC curves for bowel wall thickness (BWT) of the thickest bowel segment for (A) the IUS at 1, 3, and 6 months in predicting patients with no major therapy escalation at all time points after the IUS and achieving remission by 1 year, and (B) the intestinal ultrasound at 1 year for patients in remission vs not. Proposed pediatric BWT cut-offs based on sensitivity/specificity (Youden index) for each curve are noted.

BWT cut-offs of the thickest bowel segment to predict no therapy escalation and remission by 1 year are displayed in Figure 3. Reducing the thickest BWT to ≤2.8 mm by 6 months had good sensitivity (73%) and specificity (84%) for predicting no therapy escalation and remission by 1 year. At 1 year, the thickest BWT being ≤2.5 mm also had good sensitivity (72%) and specificity (90%) to predict remission.

### 3.6. Treatment outcomes

Seven patients (12%) required escalation of their induction therapy (either dietary or steroids) to a biologic due to inadequate treatment response to diet (e.g. exclusive enteral nutrition or Crohn’s Disease Exclusion Diet) or steroid induction. Initial maintenance therapy choices for all patients included anti-TNF (*n* = 31/61, 51%), methotrexate monotherapy (*n* = 18/61, 30%), CDED (*n* = 7/61, 11%), azathioprine monotherapy (*n* = 3/61, 5%), and 5-aminosalicylic acid monotherapy (*n* = 2/61, 3%). Seventeen of the patients who did not start with a biologic medication as their maintenance therapy (*n* = 17/30, 57%) did have to escalate to a biologic, resulting in 47 patients (*n* = 47/61, 77%) using a biologic by 1 year post-diagnosis.

**Figure 4.**
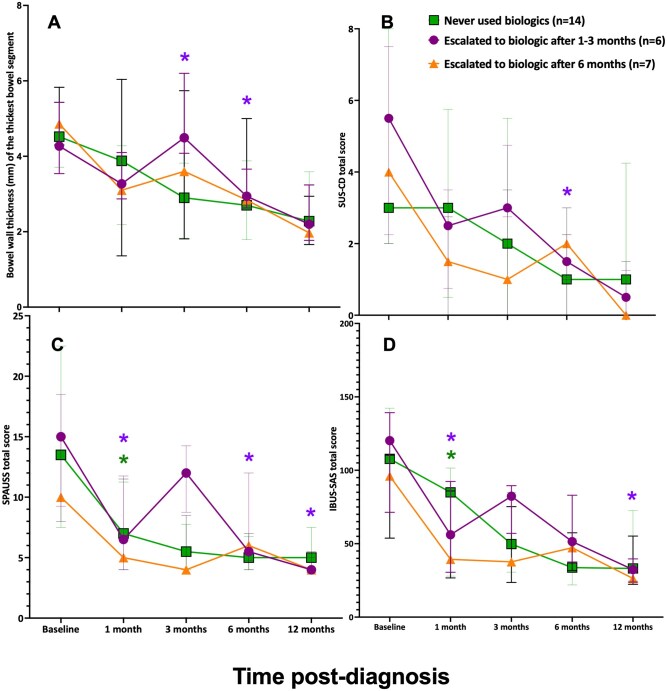
(A) Median bowel wall thickness (BWT) of the thickest bowel segment, (B) SUS-CD total score, (C) SPAUSS total score, and (D) IBUS-SAS total score over 1 year for those who never needed biologics, those who escalated to biologics after 1-3 months, and those who escalated to biologics after 6 months. Error bars represent the 25th to 75th interquartile ranges. *P < .05 was significant. SUS-CD, Simple Ultrasound Activity Score for Crohn’s Disease; SPAUSS, Simple Pediatric Activity Ultrasound Score; IBUS-SAS, International Bowel Ultrasound Segmental Activity Score.

Figure 4 displays the median IUS total scores and BWT of the most affected bowel segment over 1 year for those that never needed biologics, those who escalated to a biologic after 1-3 months, and those who escalated to a biologic after 6 months. There was continued improvement at each time point in median scores/BWT for those who never escalated to biologics. This trend was significant for SPAUSS and IBUS-SAS reduction at 1 month compared to baseline (*P* < .05). A lack of improvement or worsening scores/BWT occurred in the preceding IUS for those who escalated therapy. The time point after escalation showed improvement in all measures. For those who escalated to a biologic after 1-3 months, this trend was significant for BWT at 3 months being significantly thicker than at 1 month (*P* < .05).

### 3.7. Surgery outcomes

Seven patients (*n* = 7/61, 12%) needed surgery in the first year post-diagnosis. All patients had an ileocecal resection (*n* = 7/7), with two who also required a jejunal resection (*n* = 2/7). The timing of surgery was a median of 4 months post-diagnosis (IQR 3-6).

The seven patients who needed surgery had significantly thicker ileum BWT, higher SUS-CD, SPAUSS, IBUS-SAS ileum scores, worse endoscopic disease (with more non-traversable terminal ileum [TI] strictures), and were significantly more likely to have a TI stricture on IUS and MRE (Table 3) at baseline. There were no significant group differences in baseline wPCDAI, FCP, CRP, or ESR. Nearly all patients who required surgery (*n* = 6/7, 86%) had baseline IUS obstructive findings (luminal narrowing with upstream bowel dilatation). These obstructive findings were persistent on subsequent IUS until surgery. Baseline MRE findings of obstruction and endoscopy findings of a non-traversable TI stricture were found in fewer of these patients (*n* = 5/7, 71% and *n* = 3/7, 43% respectively).

**Table 3.** Baseline characteristics of patients requiring surgery.

Characteristic	**Surgery (*n*** = **7), median [25th to 75th IQR] or frequency (%)**	**No surgery (*n*** = **54), median [25th to 75th IQR] or frequency (%)**	*P*-value
**Age, years**	14.8 [12.3-15]	12.1 [9.8-14]	.09
**Male**	5 (71)	36 (67)	.80
**Location of disease**			
** L1 (distal ⅓ ileum ± limited cecal disease)**	3 (43)	14 (26)	.35
** L2 (colonic)**	0 (0)	11 (20)	.19
** L3 (ileocolonic)**	4 (57)	28 (52)	.79
** L4a (upper disease proximal to ligament of Treitz)**	2 (29)	26 (48)	.33
** L4b (upper disease distal to ligament of Treitz and proximal to distal ⅓ of ileum)**	3 (43)	16 (30)	.48
** Behavior of disease**			<.001*
** Stricturing only**	6 (86)	7 (13)	
** Stricturing and penetrating**	1 (14)	0 (0)	
**Perianal disease**	1 (14)	13 (24)	.57
**wPCDAI**	57.5 [47.5-65]	55 [37.5-77.5]	.82
**ESR, mm/h**	29 [25-46]	34 [16-44]	.94
**CRP, mg/L**	51 [27-60]	40 [22-80]	.81
**FCP, µg/g**	1963 [699-3266]	1539 [985-3231]	.96
**Hemoglobin, g/L**	111 [105-113]	111 [98-120]	.95
**Albumin, g/L**	29 [27-39]	29 [24-34]	.42
**Initial maintenance therapy**			.29
** Anti-TNF biologic**	7 (100)	23 (43)	
** Methotrexate**	0 (0)	20 (37)	
** Crohn’s disease exclusion diet**	0 (0)	5 (9)	
** Azathioprine**	0 (0)	4 (7)	
** Mesalamine**	0 (0)	2 (4)	
**SES-CD ileum**	12 [9-12]	4 [0-4]	<.001*
**SES-CD total**	12 [12-21]	14 [8-21]	.98
**Ileum IUS BWT, mm**	8.89 [6.12-9.71]	3.99 [2.42-4.86]	<.001*
**Ileum SUS-CD (maximum segment score 5)**	5 [4-5]	3 [0-3]	<.001*
**Ileum SPAUSS (maximum segment score 14)**	14 [7-14]	8 [2-12]	<.05*
**Ileum IBUS-SAS (no maximum segment score)**	99 [48-102]	67 [16-79]	<.01*
**IUS TI stricture^10^**	6 (86)	1 (2)	<.001*
**MRE TI stricture**	5 (71)	7 (13)	<.01*
**Endoscopy TI not traversable**	3 (43)	2 (4)	<.001*

* *P*-value < .05 was significant.

Abbreviations: wPCDAI, weighted Pediatric Crohn’s Disease Activity Index; SES-CD, Simple Endoscopic Score for Crohn’s Disease; SUS-CD, Simple Ultrasound Activity Score for Crohn’s Disease; CRP, C-reactive protein; ESR, erythrocyte sedimentation rate; FCP, fecal calprotectin; BWT, bowel wall thickness; IUS, intestinal ultrasound; MRE, magnetic resonance enterography; TI, terminal ileum.

A binary logistic regression model including baseline wPCDAI, SES-CD total score, FCP, CRP, and IUS BWT of the terminal ileum in assessing likelihood of needing intestinal surgery in the first year post-CD diagnosis was statistically significant (χ^2^(5) = 26.906, *P* < .001). Increasing IUS BWT of the terminal ileum at baseline was significantly associated with an increased likelihood of needing surgery within 1 year (OR = 5.85, 95% CI 1.29-26.47, *P* = .02). Baseline wPCDAI (OR = 1.01, *P* = .88), SES-CD total score (OR = 1.15, *P* = .34), CRP (OR = 0.94, *P* = .23), and FCP (OR = 1, *P* = 1.0) were not significantly associated.

## 4. Discussion

To our knowledge, this is one of the largest prospective longitudinal pediatric Crohn’s disease IUS cohorts with also the longest follow-up period to date. We have demonstrated that IUS, either using BWT alone or a composite IUS score (including hyperemia, bowel wall stratification, and/or inflammatory fat), improved over time with Crohn’s disease treatment. BWT alone performed similarly or better than the composite scores throughout the year in our study, which is valuable given the increasing utilization of IUS worldwide. This may be due to known higher inter-observer agreement in BWT compared to other IUS findings (eg, Doppler, fat).^16^ Improvement in IUS occurred as quickly as the first assessment at 1 month post-diagnosis for all intestinal segments, with continued improvement over 1 year. On average, a patient’s most inflamed bowel segment reduced its BWT by nearly 50% (or by over 2 mm) by 1 year of successful treatment.

Out of all patient assessment modalities (clinical, biochemical, and endoscopic), IUS had the strongest and most persistent correlation with endoscopy, which is considered the gold-standard assessment of IBD inflammation. This strong correlation persisted with both baseline diagnostic endoscopy as well as follow-up mucosal healing assessment endoscopy. IUS also correlated with clinical and biochemical markers at all time points, but not as strongly. This matches with what has previously been found in pediatric Crohn’s disease,^7–9^ particularly with symptoms not correlating well to endoscopy.^17^ This also highlights that clinical and biochemical indices do not perform as well at predicting endoscopic inflammation in isolation, and are widely encouraged to be used in combination to improve sensitivity and specificity. IUS offers a high value measure that could be considered as a priority over symptom and biochemical interpretation.

A 2022 systematic review and consensus statement defined IUS transmural remission to be a BWT of 3 mm or less (up to 4 mm in the sigmoid colon), but this is based on adult data.^18^ To date there has been no pediatric consensus.^19^ Our study showed that pediatric patients responding to treatment and in remission rapidly decrease BWT below the 3-mm cut-off 1 month post-treatment, but by 1 year those in remission have a BWT closer to 2 mm. This is in keeping with newer data that suggest pediatric patients have a thinner bowel wall than adult IBD patients, with BWT closer to 2-2.5 mm or less for non-inflamed bowel.^20–24^ It is still unknown at what age do the BWT cut-offs reflect pediatric vs adult recommendations, and if this would be in early adolescence or later teenage years.

Three months post-diagnosis was identified as an important pediatric patient assessment time point, similar to what is currently recommended based on adult data.^6^ IUS BWT and IUS scores significantly decrease for those in remission (compared to those not in remission) as soon as 3 months post-diagnosis. A lack of improving and/or worsening IUS by 3 months was also seen in patients who then needed to be escalated to biologic therapy after this time point. A previous pilot study with 13 pediatric Crohn’s disease patients also noted that bowel hyperemia and BWT significantly decreased at 3 months for all patients after receiving treatment.^25^ In our study we also found that one of the scores (SPAUSS) was also already significantly lower in those in remission as soon as 1 month, as this score is significantly higher for the most severe cases (higher specificity but lower sensitivity). Previous studies have also found IUS response as early as 2-4 weeks,^26^ with normalization of the colon on IUS by 3 months.^27^ Therefore, repeating IUS at 1 and 3 months (or at least by 3 months), is probably a valuable pediatric IBD assessment time point. Timing is also probably dependent on the rapidity of action of the chosen treatment.

Our study also identified that during the 6-month comprehensive patient assessment, IUS BWT was the only significant predictor of achieving remission by 1 year. Improving the thickest BWT to 2.8 mm or thinner by 6 months significantly predicted no subsequent treatment escalation and achieving remission by 1 year with good sensitivity (73%) and specificity (84%). This highlights the utility of repeated IUS assessment if feasible, similarly to how clinical and biochemical assessments are currently repeated in clinical practice. A recent adult Crohn’s prospective cohort also found that the IUS done at 3-6 months post-initiation of therapy reliably predicted long-term transmural and mucosal healing on infliximab.^28^ Another adult study found early intestinal ultrasound to predict endoscopic response to anti-tumor necrosis factor α therapy.^29^

Thickened bowel wall and obstructive findings can help identify those at risk of surgery. This study showed that baseline clinical and biochemical parameters were not as useful in identifying those at risk of ileocecal resection compared to imaging and endoscopy. Increased BWT of the terminal ileum on baseline IUS was significantly associated with increased need for surgery within the year, whereas baseline clinical, biochemical, and endoscopic assessments were not significantly associated. Given that IUS can be repeated much more easily than MRE and endoscopy, this is a useful tool not only for those with baseline obstructive changes, but also in those who develop this at later time points. Civitelli et al. also described a small number of pediatric patients (*n* = 4) with baseline strictures on IUS, and found that these strictures did not demonstrate any change on repeat IUS measurement despite medical therapy.^30^ IUS identification of TI/ileocecal valve stricture (bowel lumen narrowing and pre-stenotic bowel dilatation) should be considered a high-risk finding in pediatric Crohn’s patients, prompting closer follow-up, optimization of medical therapy, and potentially earlier consultations with a pediatric IBD surgeon.

Limitations of our current study include it being an unblinded study without central readers, as well as being a single center study. The rectum was also excluded as it was difficult to accurately evaluate on trans-abdominal IUS. There was a small sample size for the surgery logistic regression in our study as only seven patients underwent surgery within the 1 year. However, strengths of our study include our large pediatric IUS cohort as well as long-term follow-up time period with repeated IUS scans.

Future prospective studies should include central blinded readers of the pediatric IUS and endoscopic images if possible. Inter-user agreement and reproducibility of IUS measurements in children should also be assessed. Mixed-model analyses of longitudinal IUS, clinical, and biochemical data to assess temporal associations should also be considered. The age when BWT cut-offs transition from pediatric to adult-specific also needs to be further elucidated. Future study of potential replacement of mucosal healing follow-up colonoscopy with IUS is also of high priority in the pediatric patient population due to the invasive and burdensome nature of repeated colonoscopies under general anesthesia.

In conclusion, IUS correlated well with endoscopic inflammation over 1 year and at baseline helped identify those at higher risk of ileocecal resection within the first year of their Crohn’s disease diagnosis. Three months post-diagnosis was a particularly informative IUS time point for identifying those responding to treatment and achieving remission, and IUS at 6 months was the only significant predictor (compared to clinical or biochemical assessments) of achieving remission by 1 year. This may be due to IUS improvement more probably reflecting deeper intestinal mucosa healing than symptoms, blood work, or FCP improvement. Furthermore, a lack of improvement or worsening on two consecutive follow-up IUS scans was seen in patients who subsequently needed escalation to biologic therapy. In our pediatric study, the median BWT of the most inflamed bowel segment improved to 2 mm or less when in remission (clinical, biochemical, and endoscopic) by 1 year, which is thinner than previously reported adult IBD BWT remission cut-offs (≤3 mm). Overall, our study demonstrates the high value and accuracy of IUS in monitoring pediatric CD, even over symptoms and biochemical markers.

## Supplementary Material

jjag036_Supplementary_Data

## Data Availability

Data, analytic methods, and study materials will be made available upon request.
