# Supplementary material for: Early intestinal ultrasound findings predict remission and treatment response at 1 year in pediatric Crohn’s disease
Source: J Crohns Colitis. 2026 Mar 20;20(3):jjag036. doi: 10.1093/ecco-jcc/jjag036 (PMC13017787; doi:10.1093/ecco-jcc/jjag036)
Supplement: jjag036_Supplementary_Data [file jjag036_supplementary_data.zip › Revised_JCC Supplemental Table 1.docx]

Supplemental Tables

| **Supplemental Table 1.** Patient (n=61) clinical and biochemical parameters at all time points | | | | | |
| --- | --- | --- | --- | --- | --- |
|  | **Baseline** | **1 month** | **3 months** | **6 months** | **One year** |
| wPCDAI | 55 [40-78] | 18 [9-30]  **p<0.001*** | 8 [0-18]  **p<0.001*** | 0 [0-10]  p=0.16 | 0 [0-2]  p=0.07 |
| CRP, mg/L | 40 [23-76] | 5 [1-10]  **p<0.001*** | 2 [1-7]  **p=0.01*** | 2 [1-5]  p=0.73 | 1 [1-3]  p=0.18 |
| ESR, mm/hr | 33 [18-44] | 13 [7-21]  **p<0.001*** | 8 [5-17]  **p=0.005*** | 7 [3-12]  p=0.23 | 6 [2-10]  p=0.21 |
| FCP, mcg/g | 1549 [982-3054] | 259 [90-641]  **p<0.001*** | 254 [98-540]  **p=0.006*** | 156 [52-419]  p=0.67 | 79 [30-293]  p=0.09 |
| wPCDAI=weighted pediatric clinical disease activity index; CRP=C-reactive protein; ESR=erythrocyte sedimentation rate; FCP=fecal calprotectin  *Time point values were compared with the previous time point | | | | | |
