# Supplementary figures and images for: Early intestinal ultrasound findings predict remission and treatment response at 1 year in pediatric Crohn’s disease
Source: J Crohns Colitis. 2026 Mar 20;20(3):jjag036. doi: 10.1093/ecco-jcc/jjag036 (PMC13017787; doi:10.1093/ecco-jcc/jjag036)

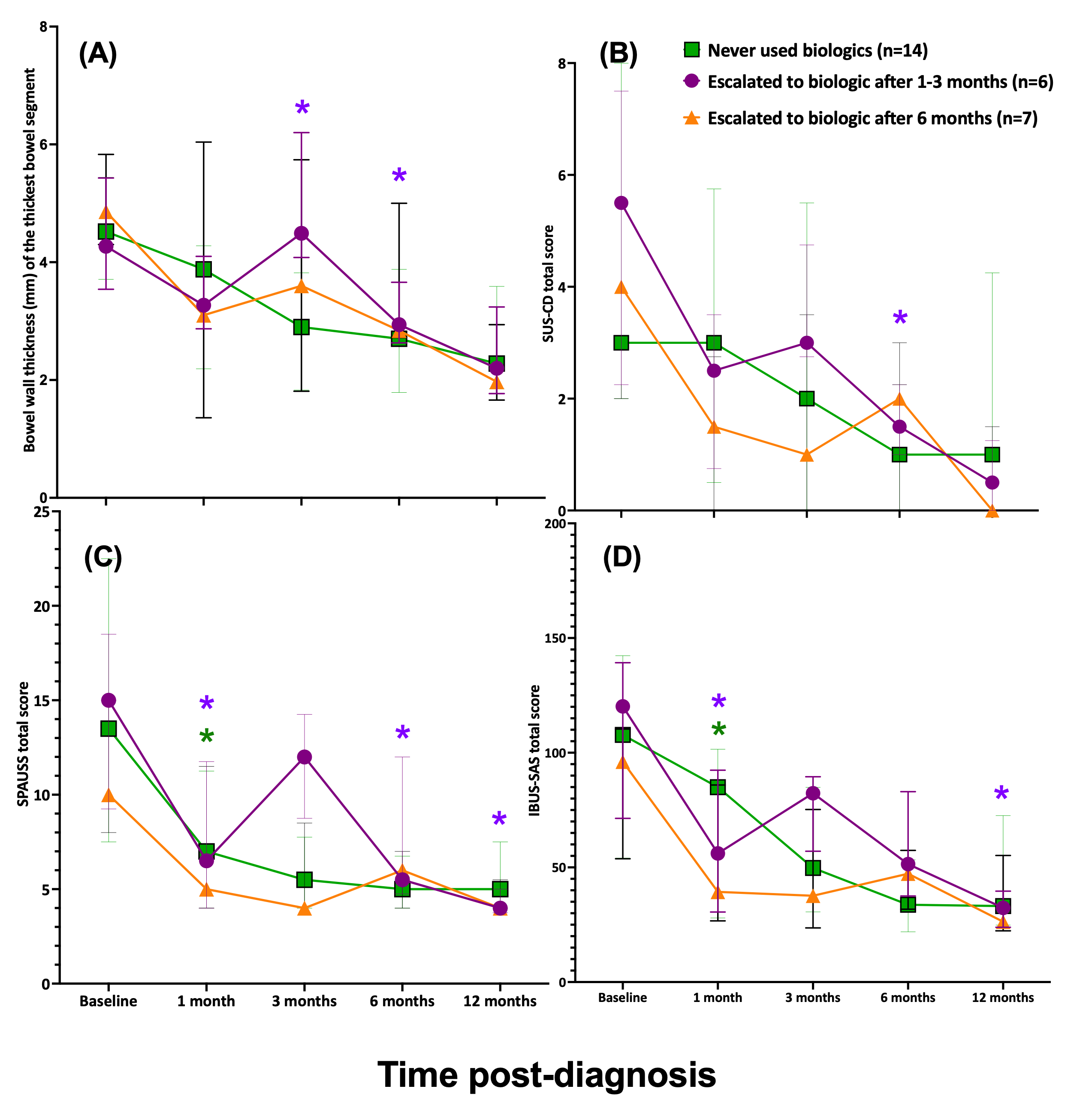

Supplement: jjag036_Supplementary_Data [file jjag036_supplementary_data.zip › SuppFig4A-D.tiff]
